# A bizarre electrocardiogram with a fruitful recovery

**DOI:** 10.1007/s12471-024-01876-6

**Published:** 2024-05-22

**Authors:** Anna van Veelen, Joëlle Elias, Pieter G. Postema, Mariëlle C. van de Veerdonk

**Affiliations:** Department of Cardiology, Amsterdam University Medical Centres, Amsterdam Cardiovascular Sciences Research Institute, University of Amsterdam, Amsterdam, The Netherlands

The electrocardiogram shows bradycardia with an extreme cardiac axis deviation and severe conduction disorders, including a right bundle branch block (RBBB)-like QRS configuration with the QRS continuing into the T wave, and absence of the ST segment, mimicking a “dying heart” phenomenon [1]. Differential diagnosis comprises (1) sodium (Na^+^) channel blockade due to intoxication with Na^+^ channel blockers (e.g. flecainide or tricyclic antidepressants), or hyperkalaemia; (2) elevated intracranial pressure; or (3) primary structural heart disease. Laboratory tests revealed hyponatraemia (131 mmol/l, normal value 135–145 mmol/l) and normal levels of potassium (4.4 mmol/l, normal value 3.4–4.9 mmol/l) and other electrolytes. Toxicology results were negative, and computed tomography showed no intracranial bleeding. Echocardiography indicated normal cardiac function despite an abnormal contraction pattern due to RBBB. The patient was transferred to the intensive care unit (ICU) for monitoring and haemodynamic support. Following rapid neurological recovery within hours, subsequent anamnesis revealed that the patient had impulsively attempted suicide by preparing a smoothie using berries and leaves from a yew tree (*Taxus baccata*).

The toxic effects of the yew tree have been known since ancient times. Its bradyarrhythmic and tachyarrhythmic effects are attributed to blockage of fast cardiac sodium channels and calcium channels [2]. Ingestion of *Taxus* plant material, whether deliberately or unintentionally, often results in fatality [3]. No effective antidote currently exists, so treatment is primarily supportive.

**Fig. 1:**
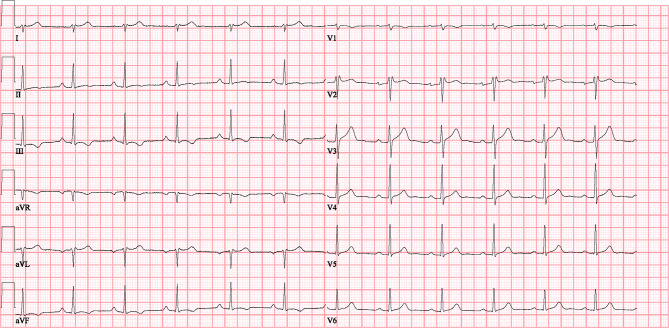
Electrocardiogram at discharge (25 mm/s, 10 mm/mV)

Our patient recovered spontaneously during the ICU stay, and vasopressors were discontinued within 6 h of hospital admission. The ECG conduction disturbances returned to normal within 12 h (Fig. 1), and the patient was discharged to the psychiatric ward for further psychological support.
